# N-myc downstream-regulated gene 1: Diverse and complicated functions in human hepatocellular carcinoma (Review)

**DOI:** 10.3892/ol.2013.1636

**Published:** 2013-10-18

**Authors:** YAN SONG, LILI CAO

**Affiliations:** Central Laboratory, Shandong Provincial Qianfoshan Hospital, Shandong University, Jinan, Shandong 250014, P.R. China

**Keywords:** N-myc downstream regulated gene-1, hepatocellular carcinoma, metastasis, differentiation, DNA damage, localization

## Abstract

N-myc downstream-regulated gene 1 (NDRG1) has been reported to be a multifunctional protein associated with carcinogenesis and tumor progression. However, the cellular function of NDRG1 remains elusive in human hepatocellular carcinoma (HCC). No NDRG1 expression is observed in normal liver tissue. Overexpression of NDRG1 has been observed in human HCC, particularly with aggressive invasion, metastasis, poor differentiation and short patient survival. In addition, recent studies have shown that NDRG1 exhibits an inhibitory effect on HCC growth *in vitro* and *in vivo*, which contrasts with previous reports indicating that NDRG1 promotes the proliferation and invasion of HCC cell lines. Further studies have shown that the localization of NDRG1 is variable, translocating to the nucleus or membrane according to the cell state, which may relate to the diverse function of NDRG1. The present study reviews our current knowledge with regard to the functions of NDRG1 in HCC and other types of human cancer.

## 1. Introduction

Human hepatocellular carcinoma (HCC) is the fifth most common malignancy worldwide and is the third most common cause of cancer-related mortality. Despite advances in surgical and non-surgical therapies for HCC, metastasis and recurrence remain the major challenges in clinical practice, and represent the most common cause of mortality in patients with HCC (1–3). Furthermore, the disease is often diagnosed at an advanced stage when conventional and effective treatment options become unavailable (4). HCC also represents a tumor type with high invasion and aggression. Recurrence of HCC following treatment remains one of the most prevalent causes leading to poor long-term survival. Thus, the ability to predict which patients have a higher risk of recurrence and a poor prognosis would help to guide surgical and chemotherapeutic treatments. Efforts have been made to predict recurrence and poor prognosis in patients with HCC following hepatectomy using clinicopathological parameters. Parameters, including tumor size, tumor number, vascular invasion, presence of satellite lesions and serum alpha-fetoprotein (AFP) level, have been reported to be useful predictors (1,5–8). With the development of molecular biology, a number of biomarkers associated with invasion, metastasis, recurrence and survival have been explored.

N-myc downstream-regulated gene 1 (NDRG1), also known as Drg1, Cap43, RTP, Rit42 and PROXY-1, is a member of the NDRG gene family. NDRG1, a 43-kDa protein with 394 amino acids, is highly conserved among multicellular organisms and is a predominantly cytoplasmic protein expressed ubiquitously in normal and neoplastic tissues (9,10). NDRG1 is involved in various aspects of carcinogenesis and development in HCC. Overexpression of NDRG1 has been observed in human HCC with aggressive invasion and metastasis, poor patient survival and poor differentiation, but not all HCC patients have shown high levels of NDRG1 (11–13). With regard to other types of cancer, a high NDRG1 expression level has shown lower invasive and metastatic potential in colorectal, prostate and pancreatic cancer (14–17). NDRG1 is a specific factor in the tumor progression of HCC.

## 2. Expression of NDRG1 in normal tissue and HCC

NDRG1 is widely expressed in the normal human body. However, in normal liver tissues, no NDRG1 expression is observed in the hepatocytes and NDRG1 is consistently positive in the biliary epithelial cells (11,18). NDRG1 expression is not affected by pathological conditions of the liver, for example, hepatitis and cirrhosis, or by different types of hepatitis virus infection. However, there have been studies showing that 6% of cirrhosis and benign liver lesions express NDRG1 (12), indicating that NDRG1 may be involved in the process of liver damage and may relate to injury severity. Studies have demonstrated that NDRG1 is significantly overexpressed in HCC compared with adjacent non-tumors and normal livers (11–13,18), which indicates that NDRG1 may be involved in hepatocarcinogenesis. Furthermore, the frequency of marked expression has been shown to be significantly higher in moderately-and poorly-differentiated HCC than in well-differentiated cases. A clinical data analysis has demonstrated that NDRG1 overexpression is correlated with a large tumor size, portal vein invasion, TNM stage, an AFP level of ≥400 U/l, intrahepatic metastasis, recurrence and poor patient survival, and that HCC patients who have positive NDRG1 expression generally have a worse prognosis than those who have no expression (11–13,18). In August 2006, a search of the GEO Profiles database revealed that NDRG1 showed significantly higher expression in non-cancerous hepatic tissue from HCC patients with venous metastasis than in patients without venous metastasis (GEO accession GDS3091) (19). Thus, it may be deduced that NDRG1 promotes the growth, development and venous metastasis of HCC. These findings indicate that the upregulation of NDRG1 may be a potentially important predictive biomarker of recurrence, metastasis and poor outcome for patients with HCC.

There have been several studies that have focused on the localization of NDRG1. Data acquired from immunohistochemistry showed that the NDRG1 protein was expressed in the cytoplasm and on the membrane of HCC cells, but not in the nucleus. An NDRG1 sequence analysis also supported these data. The protein contains a highly hydrophobic 20-amino acid sequence, which indicates that it contains either a transmembrane domain or an anchor to the cytosolic face of the lipid bilayer (12,18). The localization of NDRG1 is not invariable, but has been shown to translocate to the nucleus in response to DNA damage *in vitro*, despite lacking a nucleus localization sequence (20). It remains unknown whether the localization and relocalization of NDRG1 is associated with metastasis or recurrence.

## 3. Function of NDRG1 in oncogenesis

The silencing of NDRG1 *in vitro* has been shown to suppress the proliferation and colony formation of HCC cells. In addition, NDRG1 suppression has been found to induce an accumulation of HCC cells in the G_1_ phase and a reduction in the number of cells in the S phase, and to enhance the apoptosis and death of HCC cells. However, another study showed that the overexpression of NDRG1 in HCC cells decreased growth *in vitro* and *in vivo*, and that the majority of cells were arrested in the G_0_/G_1_ phase (21). The function of NDRG1 in the cell cycle is controversial. It has been shown that in breast and prostate cancer, NDRG1 mRNA is differentially expressed throughout the cell cycle, peaking at the G_1_ and G_2_-M phases, with lower expression in the S phase; however, the biphasic expression of NDRG1 mRNA is absent in tumor cells (20). NDRG1 phosphorylation by the protein kinase, SGK1, was found to be increased basally in p53-deficient cells and to co-localize with γ-tubulin on centromeres and to the cleavage furrow during cytokinesis (22), which indicates that NDRG1 is important in successful colon cancer mitosis. It is reasonable to assume therefore, that NDRG1 may act as a protective agent to affect tumor progression. However, whether NDRG1 functions as a tumorigenic factor or an antitumor factor remains controversial. Further research is required to clarify the function of NDRG1 in HCC growth.

NDRG1 was discovered as a differentiation-related gene (23,24), and clinical studies have shown that NDRG1 expression is significantly higher in poorly-differentiated HCC than in well- and moderately-differentiated HCCs combined (11,12). However, in pancreatic cancer, NDRG1 is highly expressed in well-differentiated cells, while the poorly-differentiated tumor cells show no NDRG1 expression (25). Increased NDRG1 has been shown to be involved in breast atypia-to-carcinoma progression (26). Furthermore, NDRG1 has shown a close correlation with β-casein or milk fat protein, which is a differentiation marker of breast tissue (27). NDRG1 also induces colon cancer cell differentiation by upregulating the expression of several colonic epithelial cell differentiation markers, namely, alkaline phosphatase, carcinoembryonic antigen and E-cadherin. There are several known cell differentiation reagents that control NDRG1 expression. These agents include ligands of peroxisome proliferator-activated receptor γ (troglitazone and BRL46593) and retinoid X receptor (LG268), and histone deacetylase inhibitors (trichostatin A, suberoylanilide hydroxamic acid and tributyrin) (14). However, the mechanisms involved have yet to be elucidated.

The downregulation of NDRG1 *in vitro* has been found to inhibit cell migration and invasion (13,28). These findings indicate that NDRG1 has potential oncogenic properties in hepatocarcinogenesis and that it contributes to HCC progression, including vascular invasion and intrahepatic metastasis. These results *in vitro* are consistent with clinicopathological data showing that NDRG1 overexpression is associated with HCC existence and progression. In gastric cancer, NDRG1 promotes tumor metastasis through epithelial-mesenchymal transition (EMT) by upregulating the expression of vimentin and Snail and downregulating the expression of E-cadherin (29). In prostate cancer, however, it has been shown that NDRG1 overexpression maintains membrane E-cadherin and β-catenin and inhibits TGF-β-induced EMT, thus inhibiting cell migration and invasion (30). NDRG1 is able to bind directly to β-catenin and E-cadherin, and the phosphorylation of NDRG1 does not interrupt the binding (31). A significant correlation has also been observed between NDRG1 and E-cadherin cell membrane localization, which may be a marker for malignant progression and a poor prognosis of prostate cancer (17). Studies have shown that NDRG1 is involved in recycling E-cadherin, thereby stabilizing it. NDRG1 recruits recycling endosomes by binding to phosphatidylinositol 4-phosphate and interacting with active membrane-bound Rab4aGTPase (32). Wnt signaling is pivotal in tumor progression and metastasis (33). Recent findings have demonstrated that NDRG1 interacts with the Wnt receptor, LRP6, blocking Wnt signaling and impairing the metastatic progression of tumor cells in prostate and breast cancer.

Furthermore, NDRG1 is able to modulate the immune response through several signaling pathways, including the complement pathway, antigen-dependent B cell activation, cytokines and the inflammatory response, and stress induction of HSP regulation, which may be correlated with tumor immunity (13). It is possible that NDRG1 is an important immune regulatory factor in HCC.

## 4. Regulation of NDRG1 during tumor progression

NDRG1 mRNA expression has been found to increase concomitantly with p53 expression, following a similar time-course in breast and prostate cancer (20). The area upstream of the transcriptional initiation site of NDRG1 contains a p53 binding site at 406 bp. It has been demonstrated that p53 binds to this site, upregulating NDRG1 expression, and that NDRG1 is necessary for p53-mediated caspase activation and apoptosis in colon cancer cells; however, p53-induced NDRG1 expression only occurs in certain cells (34). It has been shown that NDRG1 suppression sensitizes hypoxic HCC cells to doxorubicin-induced apoptosis (35). Doxorubicin is a potent activator of p53, so NDRG1 may be a direct target gene of p53 in hypoxic HCC. NDRG1 expression also induces the inhibition of intestinal epithelial cell proliferation following polyamine depletion, but the effect is decreased when p53-binding sites within the NDRG1 proximal promoter region are deleted (36). NDRG1 is able to upregulate the expression of p21, a potent cyclin-dependent kinase inhibitor, in prostate cancer and lung carcinoma cells, independently of p53. This function is obtained by downregulating ΔNp63, a dominant-negative p63 isoform that has been found to inhibit p21 transcription, and by upregulating a truncated 50 kDa MDM2 isoform (p50 MDM2), preventing p21 from proteasomal degradation (37). As NDRG1 has been identified as a metastasis suppressor gene in colon and prostate cancer, it is possible that p21 is a molecular player in its antimetastatic activity.

Hypoxia is a common characteristic and a key stimulus in the pathophysiology of numerous solid tumors, including HCC (38,39). Cell stresses induced by the microenvironment, particularly hypoxia (40,41) and reoxygenation (42), may cause these genetic changes. NDRG1 has been found to be upregulated by hypoxia *in vitro* in HCC, and hypoxia also induces the translocation of NDRG1 to the plasma membrane *in vivo*, but not under the tested conditions *in vitro*(18). The mechanisms involved are not clearly understood.

## 5. Conclusion

NDRG1 is a multifunctional protein involved in various aspects of carcinogenesis and the development of cancer, including cell proliferation, cell cycle regulation, cell differentiation, cancer metastasis and invasion. Moreover, NDRG1 exhibits tissue-specific function and regulation, and the effect between tissues may be completely opposite. In HCC, the expression and function of NDRG1 is controversial, but in prostate cancer, the gene expression in patients with lymph node or bone metastasis is significantly reduced compared with that in patients with localized prostate cancer (43). The affect of the research methods in this controversy cannot be ruled out. However, there must be certain connections between the expression and function of NDRG1 in HCC. Efforts are required to clarify the function of NDRG1 in HCC and the mechanism of its oncogenicity. It remains unknown whether NDRG1 functions as a tumorigenic factor or an antitumor factor. Further studies are required to explore the value of NDRG1 and its regulating factors as early diagnostic markers or effective targets for targeted therapy.
